# Dynamic Changes of Th1 Cytokines and the Clinical Significance of the IFN-*γ*/TNF-*α* Ratio in Acute Brucellosis

**DOI:** 10.1155/2019/5869257

**Published:** 2019-10-07

**Authors:** Guang Xu, Peng Zhang, Rongjing Dang, Yanfang Jiang, Feng Wang, Bin Wang, Mingyuan Yang

**Affiliations:** ^1^Department of Infectious Diseases, The First Hospital of Jilin University, Changchun 130021, China; ^2^Key Laboratory of Zoonoses Research, Ministry of Education, The First Hospital of Jilin University, Changchun 130021, China; ^3^Genetic Diagnosis Center, The First Hospital of Jilin University, Changchun 130021, China; ^4^Department of Cardiac Surgery, Changchun Children's Hospital, Changchun 130051, China

## Abstract

**Background:**

T-helper type 1 (Th1) cells and Th1-produced cytokines play essential roles in the immune response to foreign pathogens, such as *Brucella* spp. The aim of this study was to evaluate the dynamic changes of Th1 cells and Th1-produced cytokines in patients with acute brucellosis and their impact on clinical decision-making.

**Methods:**

Fifty-one individuals with acute brucellosis and 17 healthy subjects were enrolled in this study. The brucellosis patients were diagnosed based on clinical symptoms, laboratory tests, and clinical examination. The levels of serum gamma-interferon (IFN-*γ*) and tumor necrosis factor-alpha (TNF-*α*), along with the percentage of Th1 cells, were determined by flow cytometry bead arrays (CBA).

**Results:**

The frequency of Th1 cells, along with the levels of IFN-*γ* and TNF-*α*, was negatively correlated with the clinical parameters. The mean serum levels of IFN-*γ* and TNF-*α* and the frequency of Th1 cells were significantly higher in the brucellosis patients in comparison with the healthy subjects (*p* < 0.05). Besides, the cytokine levels were not significantly different between the positive and negative blood culture groups. IFN-*γ* levels significantly decreased from 6 months to 12 months post treatment (*p* < 0.05). However, the IFN-*γ* levels remained higher than those of the healthy subjects by 12 months post treatment (*p* < 0.05). The IFN-*γ*/TNF-*α* ratio was significantly higher in severe cases than in nonsevere cases (*p* < 0.05).

**Conclusions:**

The IFN-*γ* levels secreted by Th1 cells remain significantly higher than those of healthy subjects more than 12 months after treatment with antibiotics. This finding is different from similar studies. The IFN-*γ*/TNF-*α* ratio may be a feasible parameter for assessing clinical severity, yet further longitudinal studies of the immunization and inflammatory reaction of brucellosis are needed in larger patient populations.

## 1. Introduction

Brucellosis, also known as undulant fever, is a highly contagious bacterial infection caused by the consumption of undercooked meat and unpasteurized milk from animals infected with *Brucella* spp. [1, 2]. The disease is difficult to diagnose, as the initial symptoms are nonspecific and easily confused with other febrile diseases. Patients with brucellosis present with a history of fever, feebleness, myalgia, arthralgia, and osphyalgia. Severe cases of brucellosis may result in hematopoietic disorders and even nervous system disturbances. Treatment of brucellosis requires dual-antibiotic therapy for 6-8 weeks. Some common combination therapies include doxycycline with rifampin, doxycycline with streptomycin, and fluoroquinolone with rifampin [3].

While blood cultures are the gold standard for diagnosing brucellosis, the test suffers from high false-negative rates. In addition, the test is expensive and requires up to 10 days for confirmation. For these reasons, physicians rely on other clinical signs and tests, including the agglutination test and other nonspecific tests to diagnose the disease, including white blood cell (WBC) counts, alanine aminotransferase (ALT) levels, aspartate aminotransferase (AST) levels, platelet (PLT) counts, erythrocyte sedimentation rate (ESR), and C-reactive protein (CRP) levels. Due to the atypical symptomatic profile, the low specificity of laboratory tests, and the high rate of false-negative results with blood culture testing, brucellosis remains a difficult disease to diagnose in the clinic. As the treatment is extensive and expensive, patients must be accurately and efficiently diagnosed in a timely manner.

Once a patient becomes infected, the bacteria disturb the immune system of the host, activating several types of immune cells, including T and B lymphocytes, natural killer (NK) cells, macrophages, and neutrophils, as well as proinflammatory cytokines that can contribute to the destruction of the bacteria [4–6]. Directly after infection, naïve CD4^+^ T cells activate and differentiate into different subsets of functional T cells, such as IFN-*γ*-secreting T-helper type 1 (Th1) cells, transforming growth factor-beta 1- (TGF-*β*1-) secreting T regulatory cells (Tregs), and interleukin- (IL-) 4-secreting Th2 cells [7–9]. In response to the foreign antigen, Th1 cells produce gamma-interferon (IFN-*γ*), various interleukin (IL), and tumor necrosis factors (TNF). In addition, the immune cells induce cell-mediated immunity and play essential roles in the development of resistance. Increased levels of IFN-*γ* and TNF-*α* have been detected in the spleens and serums of mice 7 days after being infected with the *Brucella* bacteria [10, 11]. IFN-*γ* may be one of the central proinflammatory cytokines that promote resistance to *Brucella* infection. The IFN-*γ* gene is overexpressed in immune cells directly after infection in livestock animals [12, 13]. Much of these data came from experimental animal models or immune cell models cultured *in vitro*. To build upon these studies, we used human patients to assess similar queries, including how the infection affects immune factors during disease progression.

In this study, we examined the frequency of Th1 cells in the peripheral blood of brucellosis patients and healthy subjects. In addition, we measured the serum levels of IFN-*γ* and TNF-*α*, along with some common clinical indexes, including WBC, ALT, AST, PLT, ESR, and CRP. The purpose of this study was to investigate a potential relationship between Th1-released cytokine levels during infection and the clinical markers of brucellosis. We divided the patients into two groups based on their blood culture results (positive vs. negative). While both groups showed significantly higher IFN-*γ* levels in comparison to the healthy subjects, there was no difference between the two groups. Next, we studied the dynamic changes of IFN-*γ* levels in acute brucellosis and found that they were significantly higher in both treatment groups when compared with healthy subjects, even 12 months after the standard treatment. In addition, the data showed significant differences in IFN-*γ*/TNF-*α* ratios between the two patient groups with graded severity, suggesting that the IFN-*γ*/TNF-*α* ratio may be a feasible parameter for assessing clinical severity. We believe the results will assist physicians in the discovery of new strategies for improving the judgment of brucellosis progress.

## 2. Materials and Methods

### 2.1. Patients and Controls

A total of 51 patients with brucellosis and 17 age- and gender-matched healthy subjects were recruited for this study. The patients were diagnosed and treated in the Department of Infectious Disease at the First Hospital of Jilin University between March 2016 and December 2017. The diagnosis of brucellosis was confirmed using the standard agglutination test (SAT) ≥ 1 : 100, clinical signs and symptoms, and patient history (including contact with livestock). Sixteen patients had positive blood culture results. All of these patients were in the acute stage or less than one year from the onset of brucellosis. Blood samples were obtained from the healthy controls, who were without infectious diseases, inflammation, arthritis, psoriasis, hepatitis, or diabetes.

### 2.2. Laboratory Examinations

Venous blood samples were obtained from the participants for full blood cell counts and other laboratory tests. The levels of serum WBC, ALT, AST, PLT, ESR, and CRP were determined using a chemiluminescent enzyme immunoassay (Ortho Clinical Diagnostics, Raritan, NJ, USA) and radioimmunoassay (Cosmic Corporation, Tokyo, Japan) following the manufacturers' instructions.

### 2.3. Clinical Index Assay

The clinical data of the subjects enrolled in the study were collected from hospital records. These data included sex, age, and symptom analysis.

### 2.4. Isolation of Peripheral Blood Mononuclear Cells

Fresh peripheral venous blood samples were collected from the patients and healthy subjects. Peripheral blood mononuclear cells (PBMCs) were isolated using Ficoll-Paque Plus density-gradient media (Amersham Biosciences, Little Chalfont, UK) according to the manufacturer's protocol.

### 2.5. Flow Cytometry and Cytometric Bead Array

The PBMCs were harvested and stained with allophycocyanin- (APC-) H7 mouse anti-human CD4 and PerCP-Cy™5.5 mouse anti-human CD3 (BD Pharmingen, San Diego, CA, USA) for 30 min. After staining, the cells were fixed and permeabilized using the permeabilization/fixation solution kit (eBioscience, San Diego, USA) for 30 min, followed by staining with PE-Cy7-conjugated anti-IFN-*γ* (BD Biosciences, San Diego, CA, USA) for 30 min. The frequency of CD3^+^CD4^+^IFN-*γ*^+^ (Th1) T cells was determined by flow cytometry analysis using the BD FACSAria II (Becton Dickinson, Washington, DC, USA) and analyzed with the FlowJo7.6.2 software (FlowJo LLC, Ashland, OR, USA). IFN-*γ* and TNF-*α* plasma levels were determined by cytometric bead array (CBA) analysis. The serum concentrations of cytokines were quantified using the CellQuest Pro and CBA software (Becton Dickinson, Washington, DC, USA) on a FACSCalibur Flow Cytometer (Becton Dickinson).

### 2.6. Statistical Analysis

All data were expressed as mean or median values and ranges for each group of subjects. The difference between groups was analyzed using the Kruskal-Wallis *H* nonparametric test, one-way analysis of variance (ANOVA), and Newman-Keuls analyses with SPSS 22.0 (IBM, Chicago, IL, USA) software for unpaired and paired comparisons. The relationship between variables was evaluated using Spearman's rank correlation test. One-sided *p* values of less than 0.05 were considered statistically significant.

## 3. Results

### 3.1. Increased Th1 Cells and Th1 Cytokine Levels in Acute Brucellosis Patients

**Figure 1:**
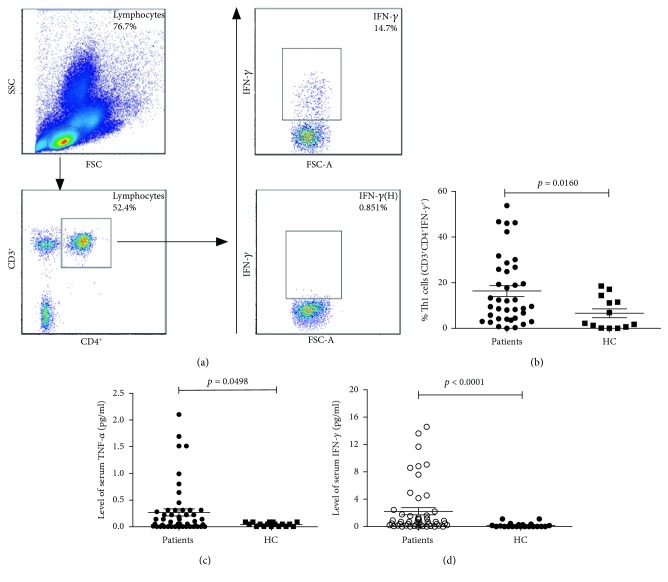
Flow cytometry analysis of the frequency of different groups of T-helper type 1 (Th1) cells. (a) Human peripheral blood mononuclear cells (PBMCs) were isolated from the participants. The cells were fixed and permeabilized, followed by staining with PerCP-Cy™5.5 mouse anti-human CD3, APC-HT mouse anti-human CD4, and intracellular staining with BV421 mouse anti-human IFN-*γ*. Next, the frequency of CD3^+^CD4^+^IFN-*γ*^+^ T cells (Th1) was calculated. (b) Flow cytometry analysis of the frequency of Th1 cells in different groups. The PBMCs from brucellosis patients were collected before treatment. Statistically significant differences were found between these two groups in three comparisons (*p* < 0.05). (c) Flow cytometry analysis of serum TNF-*α* levels in different groups. Serum tumor necrosis factor-alpha (TNF-*α*) levels were measured in patients prior to treatment. Statistically significant differences were found between these two groups in three comparisons (*p* < 0.05). (d) Flow cytometry analysis of serum gamma-interferon (IFN-*γ*) levels in the different groups. Serum IFN-*γ* levels were measured in patients prior to treatment. Statistically significant differences were found between these two groups in three comparisons (*p* < 0.05). Abbreviations: Th1: T-helper type 1; PBMC: peripheral blood mononuclear cell; IFN-*γ*: gamma-interferon; TNF-*α*: tumor necrosis factor-alpha.

Fifty-one patients in the acute stage of the disease and 17 healthy subjects were recruited. To be included in this study, the patients diagnosed with brucellosis were evaluated on three factors, including (1) clinical symptoms such as fever, feebleness, myalgia, arthralgia, osphyalgia, and profuse sweating; (2) the results of a ≥1 : 100 antibody titer to *Brucella* using the standard tube agglutination method or isolation of *Brucella* from blood bacteria cultures; and (3) no prior history of brucellosis. There were no significant differences in the distribution of age and gender between brucellosis patients and healthy subjects (Table 1). In addition to the frequency of Th1 cells, the mean serum levels of IFN-*γ* and TNF-*α* were significantly higher in the brucellosis patients when compared with the healthy subjects (*p* < 0.05; Table 1, Figure 1).

### 3.2. Correlation between the Cytokine Levels and Clinical Parameters

**Figure 2:**
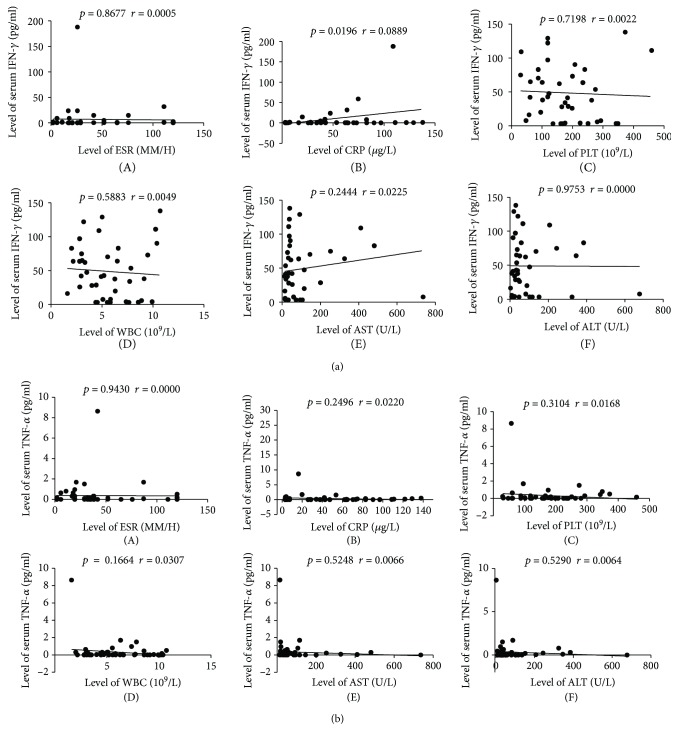
(a) The correlation analyses between IFN-*γ* and TNF-*α* levels and the clinical parameters of patients with acute brucellosis (*n* = 51). The correlation analysis charts showed no statistically significant differences (*p* > 0.05) and negative correlations between IFN-*γ* levels and several clinical parameters, including (A) erythrocyte sedimentation rate (ESR), (B) C-reactive protein (CRP), (C) thrombocyte (PLT), (D) white blood cell (WBC), (E) aspartate aminotransferase (AST), and (F) alanine aminotransferase (ALT). (b) No correlations were detected between TNF-*α* levels and the clinical parameters. Negative correlations were detected between TNF-*α* levels and the clinical indicators, including (A) ESR, (B) CRP, (C) PLT, (D) WBC, (E) AST, and (F) ALT. Abbreviations: IFN-*γ*: gamma-interferon; TNF-*α*: tumor necrosis factor-alpha; ESR: erythrocyte sedimentation rate; CRP: C-reactive protein; PLT: thrombocyte; WBC: white blood cell (leukocyte); AST: aspartate aminotransferase; ALT: alanine aminotransferase.

Through the correlation analysis, IFN-*γ* and TNF-*α* serum levels were found to negatively correlate with several clinical indicators, including WBC, ALT, AST, PLT, ESR, and CRP levels (Figure 2). Hence, IFN-*γ* levels were not an indicator of clinical manifestation and were not reflective of disease severity.

### 3.3. Comparison of Cytokine Levels between Patients with Positive Blood Culture Results and Those with a Negative Result

**Figure 3:**
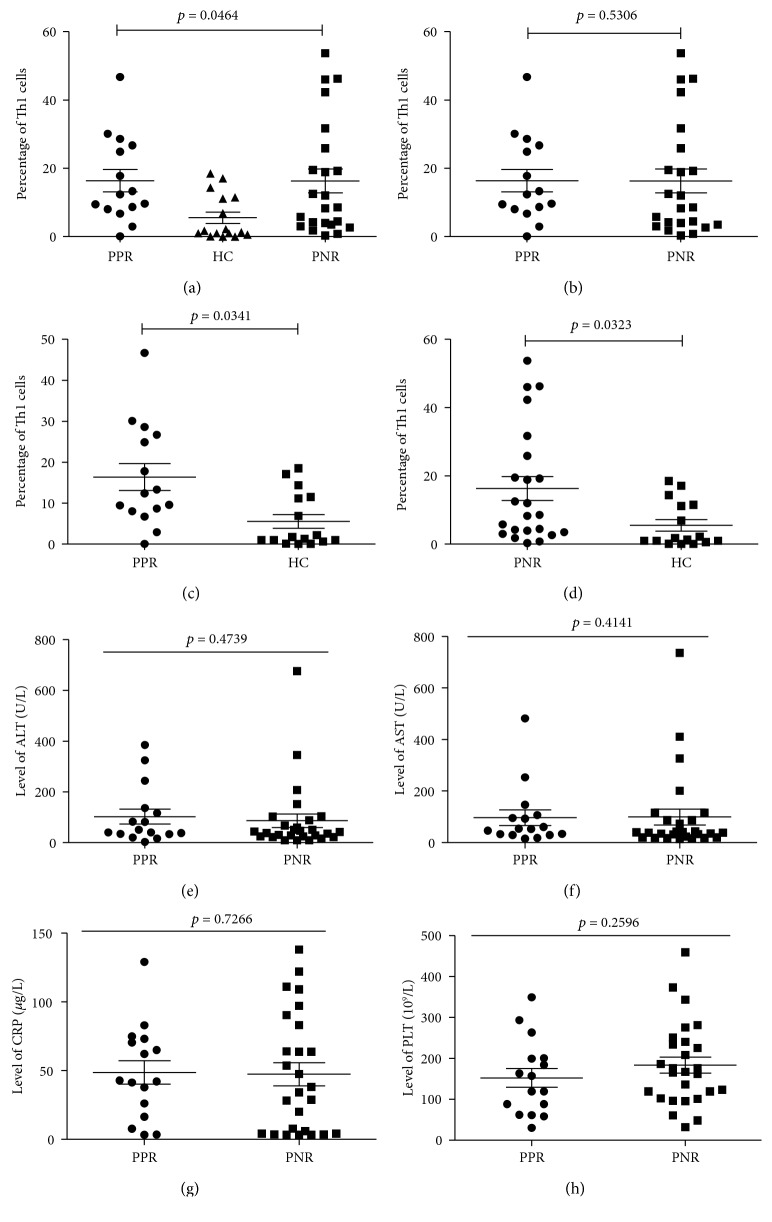
Percentages of serum Th1 cells and the clinical parameters of each group with different blood culture results. (a) Data were collected by flow cytometry and assessed with the one-way analysis of variance (ANOVA). Data analysis shows statistically significant differences in the percentages of Th1 in the different groups (*p* < 0.05). (b) Data analysis shows no statistical difference between patients with positive culture result (PPR) and patients with negative culture result (PNR) groups (*p* > 0.05). (c) Data analysis shows a statistically significant difference between PPR and HC groups (*p* < 0.05). (d) Data analysis showed a statistically significant difference between PNR and HC groups (*p* < 0.05). (e–h) The clinical parameters (ALT, AST, CRP, and PLT) in both groups with different blood culture results. No significant differences in any of the clinical parameters were detected between the two groups. Abbreviations: PPR: patients with positive culture result; PNR: patients with negative culture result; HC: healthy controls.

The patients with new-onset brucellosis were divided into two groups based on the blood culture results (Table 2). Both groups of brucellosis patients had significantly higher Th1 cell levels in comparison with the healthy subjects (*p* < 0.05, Figures 3(c) and 3(d)), while there was no significant difference in Th1 cell levels between the two groups (*p* > 0.05, Figure 3(b)). In terms of clinical parameters, there was no significant difference between the culture-negative and culture-positive groups (*p* > 0.05, Figures 3(e) and 3(f)). The Th1 cells and IFN-*γ* levels were independent of the replication and activity of brucella in the peripheral blood and still could not be used as indicators for clinical diagnosis or staging.

### 3.4. Dynamic Change Trend of IFN-*γ* Levels in the Progression of Brucellosis

**Figure 4:**
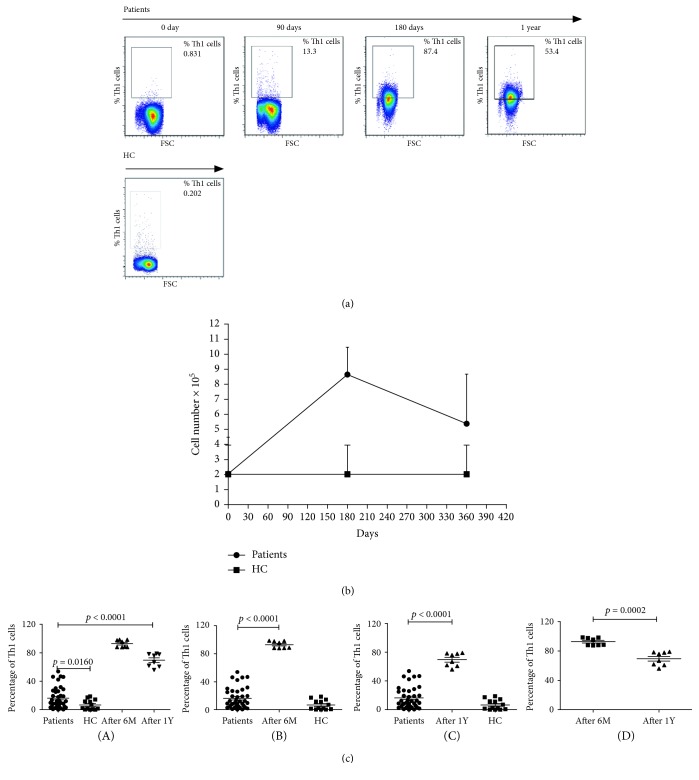
The percentage analysis of serum Th1 cells in different stages at 0, 6, and 12 months. (a) PBMCs were collected in the peripheral blood of new patients and follow-up subjects. The cells were fixed and permeabilized, followed by staining with PerCP-Cy™5.5 mouse anti-human CD3, APC-HT mouse anti-human CD4, and intracellular staining with BV421 mouse anti-human IFN-*γ*. Next, the frequency of CD3^+^CD4^+^IFN-*γ*^+^ T cells (Th1) was calculated with pictures showing IFN-*γ* in different stages. (b) Dynamic changes in Th1 cell numbers at 0, 6, and 12 months. (c) Percentage of Th1 cells in different groups. (A) Data analysis of the percentages of Th1 cells in different groups. The data are shown as the mean values of individual participants from two separate experiments. The horizontal lines indicate the median values of the different groups. A statistically significant difference was found between the patients and HC groups (*p* < 0.05). (B) Statistically significant differences could be observed in the percentages of Th1 cells between 0 and 6 months later in these two groups (*p* < 0.05). (C) Statistically significant difference could be observed on the percentages of Th1 cells between 0 and 12 months later in the two groups (*p* < 0.05). (D) A statistically significant difference was observed in the percentages of Th1 between 6 months and 12 months in the two groups (*p* < 0.05). Abbreviations: Th1: T-helper type 1; IFN-*γ*: gamma-interferon; HC: healthy controls.

During the follow-up appointments at 0, 6, and 12 months after standard treatment, four patients underwent blood work for the determination of IFN-*γ* levels. After treatment, the patients reported no uncomfortable clinical symptoms of brucellosis, and there were no cases of relapse within the first 12 months after treatment. IFN-*γ* levels at 12 months post treatment were significantly lower than those at 6 months posttreatment (*p* < 0.05; Figure 4(d)). Despite the decreasing trend in IFN-*γ* levels after treatment, the 12-month posttreatment values remained significantly higher than those of the healthy subjects (*p* < 0.05; Figures 4(a)–4(c)).

### 3.5. Differences in IFN-*γ*/TNF-*α* Ratios between Brucellosis Patients with Different Severity and Healthy Subjects

**Figure 5:**
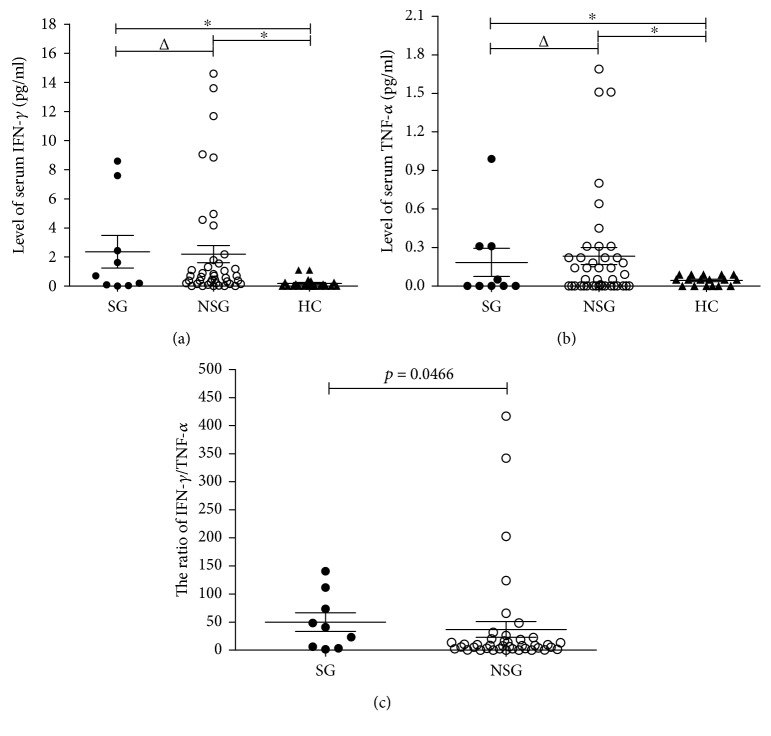
The IFN-*γ*/TNF-*α* ratios between brucellosis patients with different severities and healthy subjects. (a) IFN-*γ* plasma levels were determined by cytometric bead array (CBA) analysis, and Newman-Keuls analysis was used to evaluate the differences among groups. There were no significant differences between the severe and nonsevere groups in IFN-*γ* (*^Δ^p* > 0.05), yet significant differences were detected between the patient groups (SG and NSG) and HC in IFN-*γ* (^∗^*p* < 0.05). (b) TNF-*α* plasma levels were determined by cytometric bead array (CBA) analysis, and Newman-Keuls analysis was used to evaluate the differences among groups. There were no significant differences between the severe and nonsevere groups in TNF-*α* (*^Δ^p* > 0.05), yet significant differences were detected between the patient groups (SG and NSG) and HC in TNF-*α* (^∗^*p* < 0.05). (c) IFN-*γ*/TNF-*α* ratios for every patient were calculated and plotted for the analysis, which reveals significant differences between the severe and nonsevere groups (*p* < 0.05). Abbreviations: SG: patients in the severe group; NSG: patients in the nonsevere group; HC: healthy controls. *^Δ^p* > 0.05; ^∗^*p* < 0.05.

It has been shown that the IFN-*γ*/TNF-*α* ratio may be a useful biomarker for assessing disease severity in patients with latent tuberculosis infection [14]. In this study, there were significant differences in IFN-*γ* and TNF-*α* levels between the brucellosis patients and healthy subjects (Table 1). As previously reported [15], patients with comorbidities, such as myelitis, sacroiliitis, orchitis, encephalitis, spondylitis, and hemocytopenia, were considered as severe cases. We divided the 51 patients into severe and nonsevere groups (Table 3). The levels of IFN-*γ* and TNF-*α* were examined and found to be higher in the two treatment groups when compared with the healthy controls (Figures 5(a) and 5(b)). The IFN-*γ*/TNF-*α* ratio was assessed in both groups. More specifically, there were significant differences in the data between the severe and nonsevere groups (Figure 5(c)).

## 4. Discussion

Th1 cells and their cytokines, including IFN-*γ* and TNF-*α*, play essential roles in the biochemical responses to infectious disease pathogens and inflammation [4, 16]. They are also complicated in patients with brucellosis. Similar to the findings by Durward et al. [17, 18], we discovered that patients with brucellosis have significantly higher levels of circulating Th1 cells and cytokines. Previously, IFN-*γ* levels were shown to be increased in patients with tuberculosis [14, 19], suggesting that some intracellular bacteria, including *Brucella* spp. and *Mycobacterium tuberculosis*, display Th1-type responses during cell proliferation and cytokine production [20, 21]. In this study, we investigated the relationship between the immune reaction and several clinical indicators (WBC, ALT, AST, PLT, ESR, and CRP) in patients with brucellosis. While some negative correlations were found, these were in disagreement with previous reports [22–24]. A positive correlation was detected between inflammatory markers, ESRs, CRP levels, and cytokine levels in patients with brucellosis. In addition, some research reports have also correlated WBC, hemoglobin (HGB), and PLT with brucellosis. According to our research, these are valid indicators of inflammation but not clinical diagnosis or treatment.

When the patients were divided into two groups based on their blood culture results, the data suggested that IFN-*γ* levels and the immune reaction were unaffected by the number of bacteria present in the blood. As indicated by our findings, IFN-*γ* levels remained high in patients who were asymptomatic at the 12-month follow-up. The increased IFN-*γ* levels likely represent a memory response, indicating previous brucella infection, and may not be an indicator of active infection. In addition, patients with high peripheral blood bacterial loads were not more likely to have changes in their clinical parameters.

The Th1 cells produce a large number of serum cytokines (primarily IFN-*γ*) in the acute and chronic stages of brucellosis [22]. Previously, Rodriguez-Zapata et al. measured IFN-*γ* levels in patients with acute brucellosis, both pretreatment and three months posttreatment. IFN-*γ* levels were significantly reduced in brucellosis patients after treatment with antibiotics, reaching normal levels by three months posttreatment [23]. In another study, Ghaznavi-Rad et al. examined serum IFN-*γ* levels in both acute and chronic (symptomatic for >12 months) simultaneously and found that IFN-*γ* levels were significantly higher in patients with acute disease when compared with chronic disease [25]. However, their research did not show the variation tendency of Th1 cytokines after treatment. In this study, the dynamic trend of IFN-*γ* is different from previous reports. IFN-*γ* levels remain significantly higher than those of healthy subjects for 12 months after treatment with antibiotics (Figures 4(b) and 4(c)). Brucella infection shifted the Th1/Th2 cytokine balance, leading to the sustained elevation of IFN-*γ* levels, which is associated with the chronic progression of brucellosis. We also assumed that this trend may be related to the synergistic effect of other immune cells during brucella infection, such as Th17 and Th22 [26]. Many cytokines and chemokines are involved in the complex immune responses found in patients with brucellosis. Additional studies are needed to evaluate the change in IFN-*γ* levels and other cytokines after treatment and to evaluate the role of different immune cells on the progression of brucellosis. We aim to identify the key factors that make acute brucellosis progress to chronic disease, which may improve the clinical diagnosis and treatment of this disease.

Previously, Prabhavathi et al. found that the IFN-*γ*/TNF-*α* ratio was an effective biomarker for assessing latent tuberculosis infection [27, 28]. As an intracellular bacterium, *Brucella* may have similar biological indicators, yet we found a significant difference in IFN-*γ*/TNF-*α* ratios between the brucellosis patients and healthy subjects. A significant difference was also found between the severe and nonsevere groups. We believe this ratio may be useful as an indicator of disease severity, yet more experiments, such as immunoproteomic analyses, are needed to collect specific and nonspecific stimulus data in animal studies and verify these findings.

In summary, brucellosis patients have a higher frequency of circulating Th1 cells and higher levels of Th1 cytokines, including IFN-*γ* and TNF-*α*. The frequency of Th1 cells, along with the level of IFN-*γ* and TNF-*α*, was negatively correlated with several clinical markers, including WBC, ALT, AST, PLT, ESR, and CRP. More importantly, we found that the dynamic changes in IFN-*γ* levels were different from previous studies. It remained high in patients for more than 12 months after treatment. In the future, patients should be evaluated for extended periods (i.e., 24-36 months) to determine when the values decrease to normal levels. As in mycobacterium tuberculosis (MTB), we explored the potential use of the IFN-*γ*/TNF-*α* ratio as an indicator of disease severity, and the preliminary data confirmed this feasibility. Besides, further longitudinal studies assessing immunization and inflammatory reactions are needed in larger size population groups.

## Figures and Tables

**Table 1:** Demographics, clinical data, and levels of Th1 cells and cytokines in brucellosis patients and healthy subjects.

	Brucellosis patients (*n* = 51)	Healthy subjects (*n* = 17)
Gender (M/F ratio)	35/16^∗^	11/6
Age (years)	46.7 (20-61)^∗^	39.0 (26-54)
Agglutination test	1 : 180 (1 : 50-1 : 400)	Negative
Peripheral blood cells		
Leukocytes (WBC; ×10^9^/L)	5.69 (1.61-10.4)	6.73 (3.97-8.88)
Lymphocytes (LY; ×10^9^/L)	0.31 (0.10-0.68)	1.12 (0.41-1.77)
Platelets (PLT; ×10^9^/L)	173.0 (30.0-459.0)	214.5 (157.1-276.5)
Alanine transaminase (ALT; U/L)	83.3 (2.90-676)	16 (9-23)
Aspartate transaminase (AST; U/L)	100.4 (13.7-736.5)	18 (9-31)
Erythrocyte sedimentation rate (ESR; mm/H)	40.6 (2.00-120)	Normal
C-reactive protein (CRP; mg/L)	48.9 (3.14-138)	Normal
IFN-*γ* (pg/mL)	7.29 ± 27.3^∗^	0.13 ± 0.04
TNF-*α* (pg/mL)	0.33 ± 0.52^∗^	0.04 ± 0.04
Th1 cells (%)	32.7 ± 9.33^∗^	6.60 ± 7.02

All values, except for age and agglutination, are expressed as average (range). Values for age and the agglutination test are expressed as median (range). ^∗^*p* < 0.05 vs. healthy subjects.

**Table 2:** The demographics of two groups based on their blood culture results.

	Positive group (*n* = 16)	Negative group (*n* = 35)	*p* value
Gender (M/F)	12/4	24/11	
Age (year)	47 (30-61)^∗^	43.9 (20-57)	<0.05
Agglutination test	1 : 150 (1 : 100-1 : 400)^∗^	1 : 200 (1 : 50-1 : 400)	<0.05
Blood culture results	Positive	Negative	

^∗^Age is expressed as average (range). ^∗^Agglutination test is expressed as median (range).

**Table 3:** Patient demographics, along with immune cells and cytokine levels, in severe and nonsevere brucellosis patients and healthy control subjects.

	Severe patients (*n* = 9)	Nonsevere patients (*n* = 42)	Healthy subjects (*n* = 17)
Gender (M/F ratio)	7/2	28/14	11/6
Age (years)	49.8 (20-61)	39.0 (26-54)	39.0 (26-54)
Comorbidities			
Myelitis	*n* = 2	*n* = 0	
Sacroiliitis	*n* = 1	*n* = 0	
Orchitis	*n* = 3	*n* = 0	
Spondylitis	*n* = 2	*n* = 0	
Hemocytopenia	*n* = 1	*n* = 0	
IFN-*γ* (pg/mL)	2.36 ± 3.17^∗^	8.53 ± 29.95*^Δ^*	0.13 ± 0.04
TNF-*α* (pg/mL)	0.30 ± 0.61^∗^	1.39 ± 7.39*^Δ^*	0.04 ± 0.04
Th1 cells (%)	18.23 ± 20.12^∗^	15.85 ± 13.92^∗^	6.60 ± 7.02

Age is expressed as average (range). ^∗^*p* < 0.05 vs. healthy subjects. *^Δ^p* > 0.05 vs. healthy subjects.

## Data Availability

Our data is real, and the original data used to support the findings of this study are available from the corresponding authors upon request.
